# Gut Microbiome Modulation for Preventing and Treating Pediatric Food Allergies

**DOI:** 10.3390/ijms21155275

**Published:** 2020-07-25

**Authors:** Margherita Di Costanzo, Laura Carucci, Roberto Berni Canani, Giacomo Biasucci

**Affiliations:** 1 Department of Pediatrics and Neonatology, Guglielmo da Saliceto Hospital, 29121 Piacenza, Italy; g.biasucci@ausl.pc.it; 2 Department of Translational Medical Science-Pediatric Section, University “Federico II”, 80131 Naples, Italy; laura.carucci@outlook.it (L.C.); berni@unina.it (R.B.C.); 3 ImmunoNutritionLab-CEINGE Advanced Biotechnologies, University “Federico II”, 80131 Naples, Italy; 4 Task Force on Microbiome Studies, University of Naples “Federico II”, 80131 Naples, Italy; 5 European Laboratory for the Investigation of Food-Induced Diseases, University of Naples “Federico II”, 80131 Naples, Italy

**Keywords:** gut microbiome, food allergy, dysbiosis

## Abstract

The increasing prevalence and severity of pediatric food allergies (FA) demands innovative preventive and therapeutic strategies. Emerging evidence suggests a pivotal role for the gut microbiome in modulating susceptibility to FA. Studies have demonstrated that alteration of gut microbiome could precede FA, and that particular microbial community structures early in life could influence also the disease course. The identification of gut microbiome features in pediatric FA patients is driving new prevention and treatment approaches. This review is focused on the potential role of the gut microbiome as a target for FA prevention and treatment.

## 1. Introduction

In the past two decades, the prevalence, persistence, and severity of food allergies (FA) have been increasing [1]. This has led to an increased number of hospitalizations and costs for patients, their families, and healthcare systems [2]. Several hypotheses have been formulated to explain such phenomenon. Among these, the current “old friends and biodiversity hypotheses” propose that changes in living environment, diet, and lifestyle associated with Westernized countries have altered the microbial diversity, disrupting the immunoregulatory function of the microbiome and predisposing people to allergic sensitization [3,4,5]. The formulation of these hypotheses derives from robust evidence suggesting a key role of microbiome alteration, influenced by modern lifestyle factors, in the development of FA [6]. The purpose of this review is to present an overview of the current knowledge on the role of the gut microbiome as an innovative target of interventions against FA.

## 2. Gut Microbiome Dysbiosis and Food Allergy 

Growing evidence from human and animal studies supports a crucial role of gut dysbiosis, a state of imbalance in the gut microbial ecosystem, in FA development.

### 2.1. Evidence from Human Studies

The first studies highlighting different gut microbiome structures in subjects with FA were culture-based investigations, but this type of study was able to provide only partial results, as most of the microbiota could not be cultured [7,8]. For this reason, subsequent studies used other techniques, such as 16S rRNA sequencing and shotgun metagenomic sequencing, both based on next-generation sequencing technology, which enable more comprehensive and culture-free profiling of taxa in a given sample [9]. Unfortunately, shotgun metagenomic sequencing has not yet been widely implemented in studies with FA. Findings from 16S rRNA sequencing-based studies have shown that children with FA have a distinct gut microbiome structure compared with those without FA (Table 1). 

All studies reported in Table 1 investigated IgE-mediated FA or food sensitization. Data on gut microbiome structures in non-IgE-mediated FA are still largely unreported [20,21,22]. Interestingly, data on 46 patients affected by non-IgE-mediated cow’s milk allergy (CMA) showed dysbiosis characterized by an enrichment of *Bacteroides* (Bac 12) and *Alistipes* when compared to healthy controls, with overlapping signatures with IgE-mediated-CMA children, characterized by a progressive increase in *Bacteroides* from healthy to IgE-mediated-CMA patients. In the same study, children with non-IgE-mediated CMA had a significantly lower fecal concentration of butyrate than healthy controls [22].

The available data from human studies suggest that dysbiosis precedes FA onset. Nakayama et al. profiled the fecal bacteria compositions in allergic and nonallergic infants and correlated some changes in gut microbiome composition with allergy development in later years [23]. Azad et al. found that an increased Enterobacteriaceae/Bacteroidaceae ratio and low Ruminococcaceae abundance, in the context of low gut microbiome richness in early infancy, are associated with subsequent food sensitization, suggesting that early gut dysbiosis contributes to subsequent development of FA [11]. Moreover, the available data from human studies suggest that:

- No specific bacterial taxa could be consistently associated with FA, with a broad range of microbes that could have positive or negative influence on tolerogenic mechanisms [10,11,12,13,14,15,16,17,18,19];
- Microbiome structure early in life, particularly in the first 6 months of life, is more relevant in FA development [14];
- Dysbiosis could influence not only the occurrence, but also the disease course of FA, as suggested by different gut microbiome features comparing children who outgrow FA with patients with persistent forms of FA [14].

### 2.2. Evidence from Animal Studies

Data from animal studies provide interesting insights on the importance of gut microbiome in FA. Mice treated with antibiotics showed a predisposition to allergy development [24]. Similarly, germ-free mice do not develop immune tolerance and maintain a Th2 immune response to orally administered antigen [25,26,27]. This effect can be corrected by the reconstitution of the microbiome early in life, but not at later ages. These findings document a decisive role of the gut microbiome in the acquisition of immune tolerance to food antigens. Indeed, an early state of eubiosis allows for a change in the lymphocyte Th1/Th2 balance, favoring a Th1 cell response; while dysbiosis alters the host–microbiome homeostasis, producing a shift of the Th1/Th2 cytokine balance toward a Th2 immune response and a consequent activation of Th2 cytokines with an increased IgE production [28]. Interestingly, the gut microbiome is also able to transmit susceptibility to FA. In a mouse model susceptible to FA, because of a gain-of-function mutation in the IL-4 receptor, investigators showed that reconstitution of germ-free mice with a microbiome derived from sensitized susceptible mice, but not from sensitized resistant mice, transferred FA susceptibility to the recipient mice [29]. Other studies have shown that *Clostridium* species effectively exert an allergy-protective action in a FA mouse model, reducing allergic response [30,31]. A suppressive role of the microbiome in FA is also supported by “humanized mouse models”, created with inoculation of microbiota derived from human feces of healthy donors, resulting in reduction of allergic response [31,32]. Feehley et al. showed that germ-free mice colonized with feces from healthy donors were protected against CMA, whereas animals colonized with the feces from CMA infants showed severe anaphylactic responses to cow’s milk proteins with an increase in specific IgE response, and production of Th2 cytokines [31]. 

## 3. Gut Microbiome: Immune and Nonimmune Mechanisms of Action against Food Allergy 

### 3.1. Mechanisms of Action at the Cellular Level

The gut microbiome plays an essential role in mediating immune tolerance by promoting several immune and nonimmune mechanisms of action against FA. Current evidence suggests that the gut microbiome protects against FA, inducing the activation of T-regulatory (Treg) cells, which were found to be depleted in germ-free mice, with a consequent predisposition to FA development [33]. Microbiota-induced Treg cells express the nuclear hormone receptor RORγt and differentiate along a pathway that also leads to Th17 cells; in contrast, in the absence of RORγt in Treg cells there is an expansion of GATA-3-expressing Treg cells and conventional Th2 cells, and Th2-associated pathology is exacerbated [34]. 

The mechanisms operating in the generation of protective RORγt+ Treg cells by the commensal microbiota, including Clostridiales and Bacteroidales, is characterized by a pathway involving the Myeloid differentiation primary response 88 (MyD88); this, in turn, is an essential signal transducer of several innate immune cytokines (IL-1, IL-18, IL-33) and of Toll-like receptor signaling pathways. Deletion of *MyD88* in Treg cells abrogated the protective effect, thus establishing a MyD88–RORγt signaling axis operative in nascent Treg cells in the gut that mediates tolerance induction by the commensal microbiota in FA [35].

It has been previously established that MyD88 in Treg cells regulates the IgA response to gut microbiota and dietary antigens [36,37], which in turn plays an essential role in engendering host–microbiome symbiosis [38]. FA dysbiosis leads to disruption of the commensal microbiota–Treg cell MyD88–RORγt+ axis in FA; FA infants and mice had decreased secretory IgA binding to gut microbiota and, remarkably, increased IgE binding.

In addition to the direct effect on Treg cells, a healthy gut microbiome protects against FA by affecting enterocyte function and regulating its barrier-protective properties. Innate lymphoid cells (ILCs), which are abundant in mucosal and barrier sites, are involved in these defense mechanisms [39]. Among other factors, ILC3 produce IL-22, a cytokine of crucial importance in maintaining tissue immunity and physiology via its pleiotropic action in promoting antimicrobial peptide production, enhancing epithelial regeneration, increasing mucus production, and regulating intestinal permeability to dietary allergens [40]. 

Moreover, Feehley et al. demonstrated that mice colonized with fecal microbiota from healthy infants showed upregulation of a unique set of genes in epithelial cells of the ileum, for example *Fbp2*, which encodes the gluconeogenic enzyme fructose-bisphosphatase 1, which plays a relevant role in the maintenance of gut eubiosis. By contrast, mice colonized with fecal microbiota from CMA infants showed downregulation of *Tgfbr3* and *Ror2*, which are important for the epithelial repair [31]. 

The microbiome also promotes B-cell receptor editing within the lamina propria upon colonization [41]. Regulatory B cells have immunosuppressive capacity, which is often mediated by IL-10 secretion, but also IL-35 and TGF-β production [42]. An additional immunoregulatory role is the upregulation of IgG4 antibodies during differentiation to plasma cells [43]. 

### 3.2. Metabolic Level: Immunoregulatory Metabolites

Additional potential mechanisms by which gut microbiome exerts pro-tolerogenic effects in the gut are related to the production of immunoregulatory metabolites, which interact with the host immune cells to promote nonresponsiveness to innocuous luminal antigens [44]. 

The use of metabolomics is considered a powerful top-down biological systems approach, and it is essential to reveal the genetics–environment–health relationship, as well as the clinical biomarkers of diseases. Small-molecule metabolomics is the systematic identification, characterization, and quantification of all small metabolic products created by using specific cellular processes in a biological system. Metabolomics uses high-throughput techniques to characterize and quantify small molecules in several biofluids, such as feces, urine, plasma, serum, and saliva [45]. The metabolomic features of gut microbiota are still largely unexplored. Preliminary data available on short-chain fatty acid (SCFA) profiles are opening new perspectives for intervention. SCFAs, including acetate, propionate, valerate, and butyrate, are derived from microbial fermentation of dietary fibers in the colon [46]. SCFAs are a major energy source for colonocytes [47]. 

SCFAs directly engage G-protein-coupled receptors (GPCR) on intestinal epithelial cells (e.g., GPR41, GPR43, GPR109A, and Olfr78), or can passively diffuse through the cell membrane to inhibit histone deacetylases (HDAC) in epithelial and intestinal immune cells [48,49]. The downstream effect on enterocytes is regulation of the expression of genes involved in energy metabolism, cell proliferation and differentiation, and fortification of the epithelial barrier (tight junctions and mucus production) [50]. SCFAs also affect gut inflammatory and tissue repair processes by altering NLRP3 inflammasome and autophagy activity [51].

**Figure 1:**
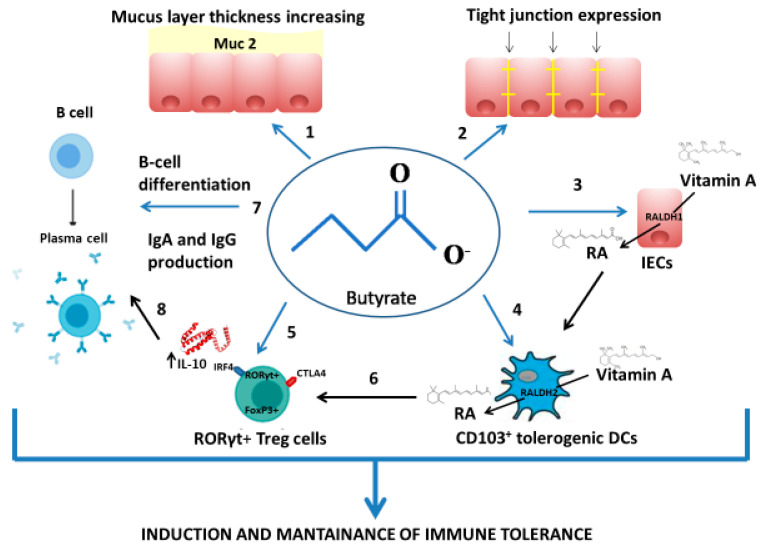
Butyrate exerts immune and nonimmune mechanisms of action against food allergy (FA). Butyrate can improve gut epithelial barrier integrity, increasing mucus layer thickness (enhancing mucin genes’ expression, in particular Muc2) (**1**) and tight junction expression (**2**). Among immune mechanisms, butyrate acts on intestinal epithelial cells (IECs) through two different pathways: (a) the inhibition of histone deacetylases (HDAC) 1 and 3 with subsequent increases in retinaldehyde dehydrogenases (RALDH) 1 activity and retinoic acid (RA) levels (**3**); (b) the interaction with G-protein-coupled receptor (GPR) 43, with subsequent increases in Vitamin A metabolism and epithelial barrier integrity. The effect of butyrate on dendritic cells (DCs) results in increasing RALDH2 activity and RA levels through direct (interaction with GPR109A expressed by DCs) and indirect (RA produced by IECs) mechanisms (**4**). Butyrate is also able to induce retinoic acid-related orphan receptor γt (RORγt)^+^ Forkhead box P3 (FoxP3)^+^ T regulatory (Treg) cells thanks to the inhibition of HDAC6 and 9, which leads to increase of FoxP3 gene expression, as well as the production and suppressive function of Treg cells (**5**). The induction of RORγt^+^ FoxP3^+^ Treg cells is also mediated by DCs interaction (**6**). Butyrate may induce B-cell differentiation and IgA and IgG production through HDAC inhibition which leads to acetylation of specific genes involved in B-cell differentiation and/or IgG and IgA production (**7**). Moreover, butyrate increases the cellular metabolism necessary for B-cell differentiation and Ig production. These mechanisms are also strongly influenced by IL-10 secreted by Treg cells (**8**). In the figure, the blue arrows indicate the direct effect of butyrate, the black arrows indicate the indirect effect of butyrate.

Among SCFAs, butyrate exerts a pivotal role in immune tolerance. It has been found that SCFAs are able to increase colonic Treg cells’ frequency, and in vitro propionate treatment of colonic Treg cells from germ-free mice significantly increases FoxP3 and IL-10 expression, a key cytokine that regulate Treg cell functions [52,53]. Similarly, it has been demonstrated that butyrate facilitates generation of activated FoxP3+ Treg cells in mouse model [54]. Butyrate is able to enhance Vitamin A metabolism, in turn inducing the activity of aldehyde dehydrogenases (ALDH) in CD103+ dendritic cells (DCs) in the gut and increasing the percentage of Treg cells and IgA production [55]. Additionally, butyrate promotes B-cell differentiation and increases IgA and IgG production [56]. The mechanisms are multiple and involve a strong epigenetic regulation of gene expression through the inhibition of HDAC [52,53,57] (Figure 1). 

Butyrate-producing bacteria represent a functional group, rather than a coherent phylogenetic group [58]. Dysbiosis results in the suppression of high-butyrate-producer species, leading to a reduction in overall butyrate production. Thus, different types of dysbiosis may share the same metabolic features, leading to similar effects in terms of butyrate or other metabolite levels that could facilitate the occurrence of FA. Starting from these data, we tested oral butyrate in a murine model of CMA and observed that it inhibited acute allergic skin response and anaphylactic symptom score, body temperature decrease, intestinal permeability increase, and beta-lactoglobulin (BLG)-specific IgE, IL-4, and IL-10 production, suggesting a protective role of butyrate against FA [59,60]. Moreover, butyrate supplementation enhanced the desensitization of effector cells induced by oral immunotherapy in a murine model of CMA, with effective reduction of mast-cell and basophil activation upon antigen challenge, and enhanced Treg cells’ functionality [61].

Besides these preliminary data derived from murine models of CMA, results from human studies have confirmed the important role of SCFAs in FA (see Section 4.4).

Metabolomics will provide important insights into not only the pathogenesis of FA, but also the disease severity. FA is associated with disease-specific metabolomic signatures, especially in sphingolipid and phospholipid metabolism, which distinguish it from asthma. Specific comparison of patients with FA and asthmatic patients revealed differences in the microbiota-sensitive aromatic amino acid and secondary bile acid metabolism. Among children with FA, the history of severe systemic reactions and the presence of multiple FAs were associated with changes in levels of tryptophan metabolites, eicosanoids, plasmalogens, and fatty acids. Lower levels of sphingolipids and ceramides and other metabolomic alterations observed in children with FA might reflect the interplay between an altered microbiome and immune-cell subsets in the gut [62]. 

The identification of bacterial metabolites that positively affect the immune tolerance network may be an interesting strategy against FA using a postbiotic approach.

## 4. Targeting Gut Microbiome in Food Allergy 

### 4.1. Environmental Factors

**Figure 2:**
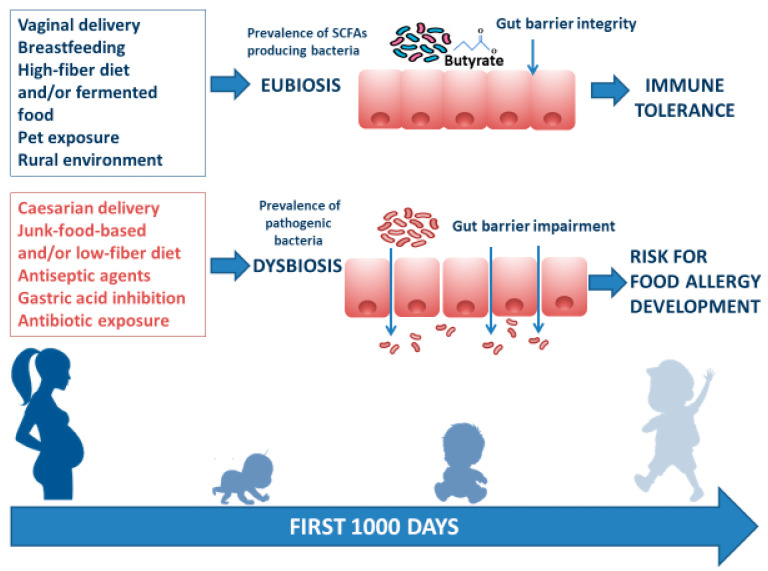
Infant gut microbiome composition and function is related to multiple environmental factors. The “first 1000 days” start from intrauterine development to the first 2 years of life and represent the frame of gut microbiome structure and function shaping. The ideal path begins with a full-term gestational period, followed by spontaneous delivery, breastfeeding provided by a mother following a Mediterranean diet lifestyle, earlier rural environmental exposure, and infant intake of a high-fiber diet and/or fermented food. All these factors are responsible for gut eubiosis, with a prevalence of SCFA-producing bacteria and gut barrier integrity, laying foundations for a healthy status and for a long-lasting protection against noncommunicable chronic diseases (such as FA) later in life. Conversely, caesarian delivery, from a mother following a junk-food-based and/or low-fiber diet, and direct or indirect childhood exposure to antiseptic agents and drugs (mainly antibiotics and gastric acidity inhibitors) leads to gut dysbiosis with prevalence of pathogenic bacteria, reduction of immunomodulatory factor production, increased gut barrier permeability, and a risk for FA development.

There are several modifiable environmental factors that can influence the occurrence of FA and can potentially be targeted to prevent FA. The window of opportunity, in which environmental factors determine an individual susceptibility to developing communicable and noncommunicable chronic diseases (including allergies) in adult life, is called the “first 1000 days”. This period goes from intrauterine development to the first 2 years of life, during which gut microbiota and immune system development are strongly influenced by environmental factors [63]. Maternal diet during pregnancy and lactation exert a direct and indirect effect on maternal gut and mammalian gland microbiota (enteromammary pathway) and play a pivotal role in early influence on infant gut microbiome composition and function [64]. Other factors such as rural environment, vaginal delivery, increased family size, exposure to pets, breastfeeding, a high-fiber diet, and/or fermented food are associated with a protective effect against FA development. In contrast, cesarean section delivery, prenatal and early-life exposure to antibiotics, gastric acidity inhibitors, antiseptic agents, and junk-food-based and/or low-fiber/high-fat diets may increase the risk of FA development. These environmental factors are mostly related to the structure and function of the gut microbiome [65,66,67,68,69,70,71,72,73,74,75,76,77,78] (Figure 2). 

### 4.2. Probiotics 

Probiotics are defined as “live microorganisms which, when administered in adequate amounts as part of food, confer a health benefit on the host” [79]. Probiotics could act at different levels in the immune tolerance network: modulating gut microbiome structure and function (e.g., increasing butyrate production) [13]; interacting with enterocytes with subsequent modulation of nonimmune (gut permeability and mucus thickness) [80,81] and immune tolerogenic mechanisms (stimulation of secretory IgA and β-defensin production) [82]; and modulation of cytokine response by immune cells [52,59,83,84,85]. Probiotic supplementation represents an interesting option to prevent and treat FA. The most common probiotic bacteria fall into two groups, namely lactobacilli and bifidobacteria. 

Recent preclinical studies on probiotic activity against FA were carried out in a murine model of egg allergy. *Lactobacillus reuteri* AB425917 restored the deteriorated profile of gut microbiota and the imbalance of Th1/Th2, inducing intestinal immune tolerance against ovalbumin-induced allergic response [86]. Song et al. isolated and identified *Lactobacillus rhamnosus* 2016SWU.05.0601, able to restore the immune imbalance of Th1/Th2 and Treg/Th17 in ovalbumin-sensitized mice by modulating gut microbiota, which contributed to the decrease in serum IgE and ovalbumin–IgE levels [87].

In a mouse model of shellfish allergy, oral administration of probiotic strain *Bifidobacterium infantis* 14.518 effectively suppressed tropomyosin-induced allergic response in both preventive and therapeutic strategies. Further results showed that *Bifidobacterium infantis* 14.518 stimulated DC maturation and CD103+ tolerogenic DC accumulation in gut-associated lymphoid tissue, which subsequently induced Treg cell differentiation aimed at suppressing Th2-biased response. The authors showed that *Bifidobacterium infantis* 14.518 regulates the alterations of gut microbiota composition. Specifically, the increase of *Dorea* and decrease of *Ralstonia* was highly correlated with Th2/Treg ratio and may contribute to alleviating tropomyosin-induced allergic responses [88].

Preclinical studies were also conducted in murine models of CMA. Neonatal monocolonization of germ-free mice by *Lactobacillus casei* BL2 modulated the allergic sensitization to cow’s milk proteins. *Lactobacillus-casei*-colonized mice developed higher casein-specific IgG responses because of casein hydrolysis by *Lactobacillus casei* into immunogenic peptides [89]. Similar results were reported by other authors who observed decreased of concentrations of IgE, IL-4, and IL-13 following administration of *Bifidobacterium infantis* CGMCC313-2 in BLG-sensitized mice [90].

Clinical studies have investigated the efficacy of selected probiotic strains against FA. The effect appears to be strain-specific. Among various probiotics, *Lactobacillus rhamnosus GG* (LGG) has emerged as a bacterial strain able to exert antiallergic actions in humans, especially in CMA. We showed that in CMA children, an extensively hydrolyzed casein formula (EHCF) supplemented with LGG induced higher tolerance rates after 6 and 12 months compared with EHCF alone and other formulas [91,92]. At the 3 year follow-up of a pediatric cohort of 220 infants with CMA, further confirmation of a greater rate of oral tolerance acquisition as well as a lower incidence of other allergic manifestations was described after treatment with EHCF+LGG compared with EHCF alone [93]. Moreover, we showed that treatment of CMA infants with EHCF+LGG resulted in the enrichment of specific strains of bacteria that are associated with higher fecal butyrate levels [13]. The World Allergy Organization guidelines consider the modulation of the immune system using functional foods a promising research hypothesis, as part of efforts to induce a tolerogenic immune environment in the context of CMA. However, the authors concluded that more evidence from randomized controlled trials is needed. They identified further research on probiotic supplementation in CMA treatment as an important area for the development of a stronger evidence base in CMA [94,95].

LGG has also been studied in patients with peanut allergies. In a clinical trial, LGG was administered with peanut oral immunotherapy for 18 months. Subjects receiving the combination treatment had higher rates of desensitization to peanut compared to placebo (82.1% vs. 3.6%, respectively) [96]. A follow-up study of 48 of the 56 children who participated in this combined probiotic and oral immunotherapy trial showed that treated individuals were more likely to have continued eating peanut compared with those who took a placebo, four years after treatment cessation (67% vs. 4%, *p* = 0.001); moreover, more participants from the treated group had smaller peanut skin-prick test size and higher peanut sIgG4:sIgE ratios compared with placebo-treated controls [97].

### 4.3. Prebiotics

A prebiotic is now defined as “a substrate that is selectively utilized by host microorganisms conferring a health benefit”, including nondigestible compounds, such as oligosaccharides or soluble fermentable fibers that are selectively utilized and promote the growth of beneficial microorganisms and improve health [98]. In particular, the galacto-oligosaccharides (GOS)/fructo-oligosaccharides (FOS) combination is the most studied. The mechanisms of action of prebiotics are due to direct and indirect effects. Indirect effects include selective fermentation, increasing populations of resident health-promoting microorganisms of the gut. SCFAs mediate prebiotics’ direct beneficial effects at the intestinal and extraintestinal level [46,99]. The supplementation of prebiotics has been proposed as a possible method of intervention in the prevention of allergic disorders [100]. However, the vast majority of the systematic reviews and meta-analyses conducted in this area have concluded that although several studies show a positive effect of prebiotics on allergic manifestations, the existing evidence is not sufficient to recommend prebiotic as a routine method for allergy prevention in formula-fed infants [101,102]. Thus, further rigorous studies in this field are required. 

### 4.4. Postbiotics

The term postbiotic refers to the use of nonviable cells or cell fractions which, when administered in adequate amounts, confer a health benefit to the host. Additionally, the term postbiotic is also related to soluble components such as SCFAs, vitamins, bacteriocins, organic acids, enzymes, hydrogen peroxide, ethanol, diacetyl, peptides, cell-surface proteins, teichoic acids, peptidoglycan-derived muropeptides, endo- and exopolysaccharides, lactocepins, plasmalogens, polyphosphates, and quorum-sensing molecules produced by live probiotic cells in fermentation processes or synthetically produced in a laboratory [103,104]. The immunomodulatory mechanisms elicited by SCFAs represent one of the strongest connections between diet, gut microbiome, and allergic diseases [44]. In a human cohort of 301 1-year old children, significant associations were reported between the composition of dietary intake and stool SCFA content, suggesting that diet can be used to modulate microbial production of SCFAs. The authors also investigated the role of SCFAs in allergy prevention and found that the children with the highest levels of butyrate had a reduced risk of becoming sensitized to food allergens [105]. As we said above, preclinical studies have shown that among SCFAs, butyrate contributes to protection against the development of FA through multiple tolerogenic mechanisms. In human observational studies, butyrate deficiency was observed in allergic children [106], whereas an enrichment of butyrate-producing taxa (Clostridia class and Firmicutes phylum) was observed in children with faster CMA resolution [14]. More recently, Cait et al., using shotgun sequencing, analyzed the fecal microbiomes (at 3 month and 1 year stool samples) of 105 atopic children from the Canadian Healthy Infant Longitudinal Development (CHILD) study to investigate whether bacterial butyrate production in the early-infancy gut is protective against the development of atopic diseases later in life. The authors found that bacteria involved in butyrate production were rather depleted in 3-month-old infants who later developed atopy. Analyzing the gut microbiome function, they also found that 3-month-old infants who later had allergic manifestations lacked genes encoding key enzymes for both carbohydrate breakdown and butyrate production [107]. We evaluated the direct effects of butyrate on peripheral blood mononuclear cells (PBMCs) from children affected by challenge-proven IgE-mediated CMA. PBMCs were stimulated with BLG in the presence or absence of butyrate. Preliminary results show that butyrate stimulates IL-10 and IFN-γ production and decreases the DNA methylation rate of two cytokine genes. 

These data suggest the potential of a postbiotic approach, based on the use of SCFAs against FA. However, clinical trials based on SCFA supplementation for FA prevention and treatment have yet to be undertaken. Therefore, there is no current recommendation from any scientific society on the optimal postbiotic administration frequency for the prevention and treatment of FA.

### 4.5. Synbiotics

Synbiotics are a mixture of prebiotics and probiotics that affect the host by improving the survival and implantation of live microbial dietary supplements in the gastrointestinal tract, improving the health of the host [108]. Candy et al. [109] designed a study to investigate whether synbiotic ingredients could improve the gut microbiota in infants with non-IgE-mediated CMA to achieve a microbial composition close to that seen in healthy, breastfed infants. Infants with suspected non-IgE-mediated CMA were administered the test formula containing the synbiotics, or a control formula without the synbiotics. The test formula was a hypoallergenic, nutritionally complete amino-acid-based formula, including a prebiotic blend of fructo-oligosaccharides and the probiotic strain *Bifidobacterium breve* M-16V. The control formula was an amino-acid-based formula without synbiotics. The authors concluded that the amino-acid-based formula containing specific synbiotics improved the fecal microbiota of infants with suspected non-IgE-mediated CMA, approximating the composition of the gut microbiota of healthy, breastfed infants. 

Interestingly, *Bifidobacterium breve* M-16V may alter the gut microbiota to alleviate allergy symptoms by IL-33/ST2 signaling. These results indicated that gut microbiota is essential for regulating FA to dietary antigens, and demonstrated that intervention in bacterial community regulation may be therapeutically related to FA [110].

However, although these preliminary data are promising, further studies are needed to evaluate the efficacy of this approach on clinical symptoms.

A planned but not yet recruiting randomized, double-blind clinical trial of children at high risk for allergy will compare partially hydrolyzed infant formula with synbiotics vs. standard infant formula (NCT03067714) for the primary outcome of doctor-diagnosed IgE-mediated allergic manifestations.

### 4.6. Fecal Microbiota Transplantation

Fecal microbiota transplantation represents another approach to shape the gut microbiota in FA patients. The idea behind this strategy is that fecal microbiota transplantation from a healthy donor to a disease recipient can restore gut eubiosis by promoting oral tolerance [111,112]. Recently, a human study revealed that fecal microbiota transplantation is able to induce remission of infantile allergic colitis through restoration of gut microbiota diversity [113]. However, the available data in this field remain limited and the relevant scientific work is just beginning. A small Phase I open-label trial to evaluate the safety and efficacy of oral encapsulated fecal microbiota for the treatment of peanut allergy is underway (NCT02960074).

## 5. Conclusions and Future Perspectives

Growing evidence suggests that dysbiosis in early life is a crucial factor for FA development. For this reason, the gut microbiome is emerging as an innovative target for pediatric FA prevention and treatment [114,115,116,117,118,119]. Shaping the gut microbiome with an intervention in the form of modifiable environmental factors and/or with pro-/pre-/syn-/postbiotics is a promising strategy against FA. In this field, evidence from human and animal studies is encouraging, but many questions remain unresolved.

## Figures and Tables

**Table 1:** Main gut microbiome differences in 16S-rRNA-sequencing-based studies between pediatric patients with and without FA.

	Food Allergy	OTUs	Diversity	Main Features Associated with Food Allergy
Ling et al. 2014 [10] (*n* = 34; FA)	Cow’s milk, egg, wheat, nut, peanuts, fish, shrimp, soybean	↓	=	↑ Bacteroidetes ↑ Proteobacteria ↑Actinobacteria ↓ Firmicutes
Azad et al. 2015 [11] (*n* = 12; FS)	Cow’s milk, egg, peanut	↓	=	↓ Enterobacteriaceae ↓ Bacteroidaceae
Chen et al. 2015 [12] (*n* = 23; FS)	Egg white, cow’s milk, wheat, peanut, soybean	N.R.	↓	↑ Firmicutes ↑ Proteobacteria ↑ Actinobacteria ↓ *Veillonella*
Berni Canani et al. 2016 [13] (*n* = 39; FA)	Cow’s milk	↑	N.R.	↑Ruminococcaceae ↑ Lachnospiraceae ↓Bifidobacteriaceae ↓Streptococcaceae ↓Enterobacteriaceae
Bunyavanich et al. 2016 [14] (*n* = 226; FA)	Cow’s milk	↑	N.R.	↑ Bacteroidetes ↑*Enterobacter*
Inoue et al. 2017 [15] (*n* = 4; FA)	Egg, wheat, soybean, sesame, cow’s milk, peanut, shrimp, crab	N.R.	N.R.	↑ *Lachnospira* ↑ *Veillonella* ↑ *Sutterella* ↓ *Dorea* ↓ *Akkermansia*
Kourosh et al. 2018 [16] (*n* = 68; FA)	Tree nuts, fish, milk, egg, sesame, soy	↑	N.R.	↑*Oscillobacter valericigenes* ↑*Lachnocrostidium bolteae* ↑ *Faecalibacterium * sp.
Fazlollahi et al. 2018 [17] (*n* = 141; FA)	Egg	N.R.	N.R.	↑ Lachnospiraceae ↑ Streptococcaceae ↑ Leuconostocaceae
Dong et al. 2018 [18] (*n* = 60; FA)	Cow’s milk	N.R.	↓	↑ Lactobacillaceae ↓ Bifidocacteriaceae ↓ Ruminococcaceae
Savage et al. 2018 [19] (*n* = 14; FA)	Cow’s milk, egg, wheat, soy, walnut, peanut	=	=	↓ *Citrobacter* ↓ *Oscillospira* ↓ *Lactococcus* ↓ *Dorea*

FA: food allergy; FS: food sensitization; OTUs: operational taxonomic units; N.R.: not reported; ↑: increase; ↓: decrease; =: unchanged.

## Abbreviations

ALDH	Aldehyde dehydrogenases
BLG	Beta lactoglobulin
CMA	Cow’s milk allergy
DCs	Dendritic cells
EHCF	Extensively hydrolyzed casein formula
FA	Food allergy
FoxP3	Forkhead box P3
FOS	Fructo-oligosaccharides
Fbp2	Fructose-Bisphosphatase 2
GATA-3	GATA-binding protein 3
GOS	Galacto-oligosaccharides
GPCRs	G-protein coupled receptors
HDAC	Histone deacetylases
IECs	Intestinal epithelial cells
IFNγ	Interferon gamma
IL	Interleukin
ILC	Innate lymphoid cells
LGG	Lactobacillus rhamnosus GG
NLRP3	NOD-, LRR- and pyrin domain-containing protein 3
OTUs	Operational taxonomic units
PBMCs	Peripheral blood mononuclear cells
RA	Retinoic acid
RALDH	Retinaldehyde dehydrogenases
RORγt	Retinoic acid-related orphan receptor γt
Ror2	Receptor Tyrosine Kinase Like Orphan Receptor 2
SCFAs	Short-chain fatty acids
TGFβ	Transforming growth factor-beta
Tgfbr3	Transforming Growth Factor Beta Receptor 3
Th	T helper
Treg	T regulatory
